# Supplementary material for: LGI3/2–ADAM23 interactions cluster Kv1 channels in myelinated axons to regulate refractory period
Source: J Cell Biol. 2023 Feb 24;222(4):e202211031. doi: 10.1083/jcb.202211031 (PMC9997507; doi:10.1083/jcb.202211031)
Supplement: Table S1 — lists all antibodies, reagents, primers, and plasmids as well as software and hardware used in this study. [file JCB_202211031_TableS1.docx]

Table S1. Key resources

| REAGENT or RESOURCE | SOURCE | IDENTIFIER |
| --- | --- | --- |
| **Antibodies** | | |
| Caspr (Rabbit IgG) | Elior Peles (Peles et al., 1997) | N/A |
| Caspr (Rabbit) | Abcam | ab34151, RRID: AB_869934 |
| Kv1.1 (Rabbit) | Meijer lab | N/A |
| Kv1.1 (Mouse IgG2a) | Neuromab | Clone K20/70R, RRID: AB_2750656 |
| Kv1.2 (Mouse IgG2b) | Neuromab | Clone K14/16 RRID: AB_2877295 |
| Lgi2 (Rabbit) | Atlas Antibodies | HPA017140, RRID: AB_1852833 |
| Lgi3 (Rabbit) | Proteintech | 21919-1-AP, RRID: AB_10838807 |
| Caspr2 (Rabbit) | Genscript | A01426-100, RRID: AB_1720782 |
| Tag1 (Goat) | R&D Systems | AF4439, RRID: AB_2044647 |
| Adam22 (Rabbit) | ThermoFisher Scientific | PA5-30639, RRID: AB_2548113 |
| Adam23 (Chicken) | Meijer lab | N/A |
| bIVspectrin (Rabbit) | Matthew Rasband (Yang et al., 2004) | N/A |
| PSD95 (Mouse IgG2a) | Neuromab | Clone K28/43, RRID: AB_2292909 |
| P0 (Chicken) | Aves Labs | RRID: AB_2313561 |
| V5 (Mouse IgG1) | Absea Biotechnology | K05002M05C05C |
| V5 (Mouse IgG2a) | Absea Biotechnology | K05002M10D11C |
| Goat anti-Mouse IgG1 Alexa 488 | ThermoFisher | A21121, RRID: AB_2535764 |
| Donkey anti-Rabbit IgG Alexa 488 | Jackson ImmunoResearch | 711-545-152, RRID: AB_2313584 |
| Goat anti-Mouse IgG2a Alexa 555 | ThermoFisher | A21137, RRID: AB_2535776 |
| Goat anti-Mouse IgG2b Alexa 555 | ThermoFisher | A21147, RRID: AB_2535783 |
| Donkey anti-Rabbit Alexa 555 | ThermoFisher | A31572, RRID: AB_162543 |
| Goat anti-Mouse IgG1 Alexa 647 | ThermoFisher | A21240, RRID: AB_2535809 |
| Goat anti-Mouse IgG2a Alexa 647 | ThermoFisher | A21241, RRID: AB_2535810 |
| Donkey anti-Rabbit Alexa 647 | Jackson ImmunoResearch | 711-605-152, RRID: AB_2492288 |
| Donkey anti-Chicken Alexa 647 | Jackson ImmunoResearch | 703-605-155, RRID: AB_2340379 |
| Goat anti-Mouse HRP | ThermoFisher | A16066, RRID: AB_2534739 |
| Goat anti-Human IgG HRP | Abcam | 6858, RRID: AB_955433 |
| Goat anti-Rabbit HRP | ThermoFisher | A16096, RRID: AB_2534770 |
| Goat anti-Chicken HRP | ThermoFisher | A16054, RRID: AB_2534727 |
| Rabbit anti-Goat HRP | ThermoFisher | A16136, RRID: AB_2534807 |
| **Bacterial and virus strains** | | |
| TOP10 | ThermoFisher | C404010 |
| **Biological samples** |  |  |
| **Chemicals, peptides, and recombinant proteins** | | |
| PCR buffer | Promega | D2391 |
| Phire HS II polymerase | Thermofisher Scientific | F124L |
| Dulbecco's Modified Eagle Medium (DMEM) | Gibco, Life technologies | 11965084 |
| Foetal Bovine serum | Gibco, Life technologies | 10500064 |
| Penicilin-Streptomycin mix | Gibco, Life technologies | 15140122 |
| Dulbecco's Phosphate-buffered Saline (DPBS) | Gibco | 14190094 |
| TrypLE express enzyme | Gibco | 12604013 |
| DMSO | Sigma-Aldrich | D2650 |
| Polyethyleneimine – PEI | Sigma-Aldrich | 408727 |
| Protease Inhibitor cocktail | Sigma-Aldrich | P2714 |
| Benzonase | Scientific Laboratory Supplies | E1014-25KU |
| PMSF | Cell Signalling Technologies | 8553 |
| Methanol-free Paraformaldehyde (16%) | ThermoFisher Scientific | 28908 |
| Bolt LDS sample buffer (x4) | ThermoFisher Scientific | B0007 |
| Bolt antioxidant | ThermoFisher Scientific | BT0005 |
| NP-40 | ThermoFisher Scientific | 28324 |
| Tween-20 | Sigma-Aldrich | 9005-64-5 |
| Sample Reducing Agent (x10) | ThermoFisher Scientific | B0009 |
| Colour pre-stained protein standard | New England BioLabs | P7719S |
| MOPS SDS Running Buffer (x20) | ThermoFisher Scientific | NP0001 |
| Bolt transfer buffer (20x) | ThermoFisher Scientific | BT00061 |
| KLP Milk Blocking Buffer | SeraCare | 5140-0011 |
| SuperSignal™ West Pico PLUS Chemiluminescent Substrate | ThermoFisher | 34580 |
| NP-40 | ThermoFisher | 28324 |
| Triton X-100 | Sigma-Aldrich | T8787 |
| β-Glycerophosphate | Sigma-Aldrich | G9422 |
| Deoxycholate | Sigma-Aldrich | D6750 |
| SDS | Sigma-Aldrich | 75746 |
| KCl | Sigma-Aldrich | P3911 |
| NaH_2_PO_4_ | Sigma-Aldrich | 71500 |
| NaHCO_3_ | Sigma-Aldrich | S8875 |
| Glucose | Sigma-Aldrich | G7528 |
| Sucrose | Sigma-Aldrich | S0389 |
| MgCl_2_ | Sigma-Aldrich | M2670 |
| CaCl_2_ | Sigma-Aldrich | C5670 |
| NaCl | Sigma-Aldrich | S7653 |
| NMDG | Sigma-Aldrich | M2004 |
| HEPES | Sigma-Aldrich | H3375 |
| Sodium L-absorbate | Sigma-Aldrich | 11140 |
| Thiourea | Sigma-Aldrich | T8656 |
| Sodium pyruvate | Sigma-Aldrich | P2256 |
| MgSO_4_ | Sigma-Aldrich | M2643 |
| 4-AP | Sigma-Aldrich | 275875 |
| **Critical commercial assays** | | |
| Pierce Protein Assay (BCA) | ThermoFisher Scientific | 23225 |
| Nickle-charged Ni-NTA agarose beads | Qiagen | 30230 |
| Protein A (ProtA) agarose beads | Repligen | CA-PRI-0100 |
|  | | |
| **Experimental models: Cell lines** | | |
| HEK293T | ATCC | CRL-3216 |
| **Experimental models: Organisms/strains** | | |
| Mouse: C57BL/6J | The Jackson Laboratory | Stock# 00064 |
| Adam23^tm1.1Mejr^ | Kegel et al., 2014 | MGI:5613204 |
| Adam23^tm1.2Mejr^ | Kegel et al., 2014 | MGI:5613205 |
| Lgi2 | Dies Meijer | This study |
| Lgi3^tm1.2Mejr^ | Marafi et al., 2022 | MGI: 7331636 |
| FVB(Cg)-Tg(Dhh-cre)1Mejr/J | Jeagle et al., 2003, The Jackson Laboratory | RRID:IMSR_JAX:012929 Strain #:012929 |
| Pvalbtm1(cre)Arbr | Hippenmeyer et al., 2013, The Jackson Laboratory | MGI:3590684, RRID:IMSR_JAX:017320, Strain #:017320 |
| Tg(Avil-icre/ERT2)AJwo | Lau et al., 2011, The Jackson Laboratory | RRID:IMSR_JAX:032027, Strain#:032027 |
| **Oligonucleotides** | | |
| Primer: Adam23, fw CTTCCCACTGTCATCCCACT | Integrated DNA Technologies | N/A |
| Primer: Adam23, rev CATCCCAGGCAGCATCAAAA | Integrated DNA Technologies | N/A |
| Primer: Adam23, rev AGGGCTATAGTTGCTGGCAA | Integrated DNA Technologies | N/A |
| Primer: Lgi2, fw CAG CAC CGA TGA ATT TCC TCT T | Integrated DNA Technologies | N/A |
| Primer: Lgi2, rev GAG ATG GGG ATT TGT AAC TGG C | Integrated DNA Technologies | N/A |
| Primer: Lgi2, rev GGA GTA ACC CCT CTA CTT CCA G | Integrated DNA Technologies | N/A |
| Primer: Lgi3, AACTCCAACTGTGGCCGTGCAG | Integrated DNA Technologies | N/A |
| Primer: Lgi3, CCTGTGAGTGTCTACCTAGCC | Integrated DNA Technologies | N/A |
| Primer: Lgi3, rev GGCAGGAGTCTGGTCCATGC | Integrated DNA Technologies | N/A |
| Primer: PV-Cre (KI), fw AAA TGC TTC TGT CCG TTT GC | Integrated DNA Technologies | N/A |
| Primer: PV-Cre (WT), fw CAG AGC AGG CAT GGT GAC TA | Integrated DNA Technologies | N/A |
| Primer: PV-Cre (KI), rev ATG TTT AGC TGG CCC AAA TG | Integrated DNA Technologies | N/A |
| Primer: PV-Cre (WT), rev AGT ACC AAG CAG GCA GGA GA | Integrated DNA Technologies | N/A |
| Primer: Advil, fw CCCTGTTCACTGTGAGTAGG | Integrated DNA Technologies | N/A |
| Primer: Advil, rev AGTATCTGGTAGGTGCTTCCAG | Integrated DNA Technologies | N/A |
| Primer: Cre, rev GCGATCCCTGAACATGTCCATC | Integrated DNA Technologies | N/A |
| **Recombinant DNA** | | |
| pcDNA-Adam23γ_V5/His | Meijer lab | N/A |
| pcDNA3.1-Adam23γ_Fc | Meijer lab | N/A |
| pSCT-Adam22ED_Fc | Meijer lab | N/A |
| pSCT-Caspr2ED_Fc | Meijer lab | N/A |
| pCX-Nfsc155_HA/Fc | Meijer lab | N/A |
| pcDNA3.1 Lgi3_V5/His | Meijer lab | N/A |
| pSCT-PLDM2 | Meijer lab | N/A |
| **Software and algorithms** | | |
| GraphPad Prism 9 | GraphPad Softward, Inc | N/A |
| ImageJ (Fiji) | Schindelin et al., 2012 | N/A |
| Affinity Designer | Affinity | Affinity.serif.com |
| Adobe Illustrator | Adobe | N/A |
| pClamp | Molecular Devices | N/A |
| Zen 2012 | Carl Zeiss | N/A |
| **Other** | | |
| SuperFrost Plus adhesion microscope slides | Epredia | 12312148 |
| Sample Grinding Kit | GE Healthcare | GE80-6483-37 |
| Mini gel tank and blot module set | ThermoFisher Scientific | NW2000 |
| Nitrocellulose blotting membranes | Amersham | GE10600002 |
| 4%–12% Bis-Tris mini protein gel | ThermoFisher Scientific | NP0321BOX |
| AxioImager-Z1 microscope with ApoTome | Carl Zeiss | 29700 |
| OdysseyFc Imaging System | LI-COR | N/A |
| SpectraMax Microplate reader | Molecular Devices | N/A |
| ISO-flex Stimulus Isolator | A.M.P.I. | N/A |
| ER-1 Differential Extracellular Amplifier | Cygnus Technologies | N/A |
