# Supplementary figures and images for: LGI3/2–ADAM23 interactions cluster Kv1 channels in myelinated axons to regulate refractory period
Source: J Cell Biol. 2023 Feb 24;222(4):e202211031. doi: 10.1083/jcb.202211031 (PMC9997507; doi:10.1083/jcb.202211031)

Source data Figure 2A Kozar-Gillen et al

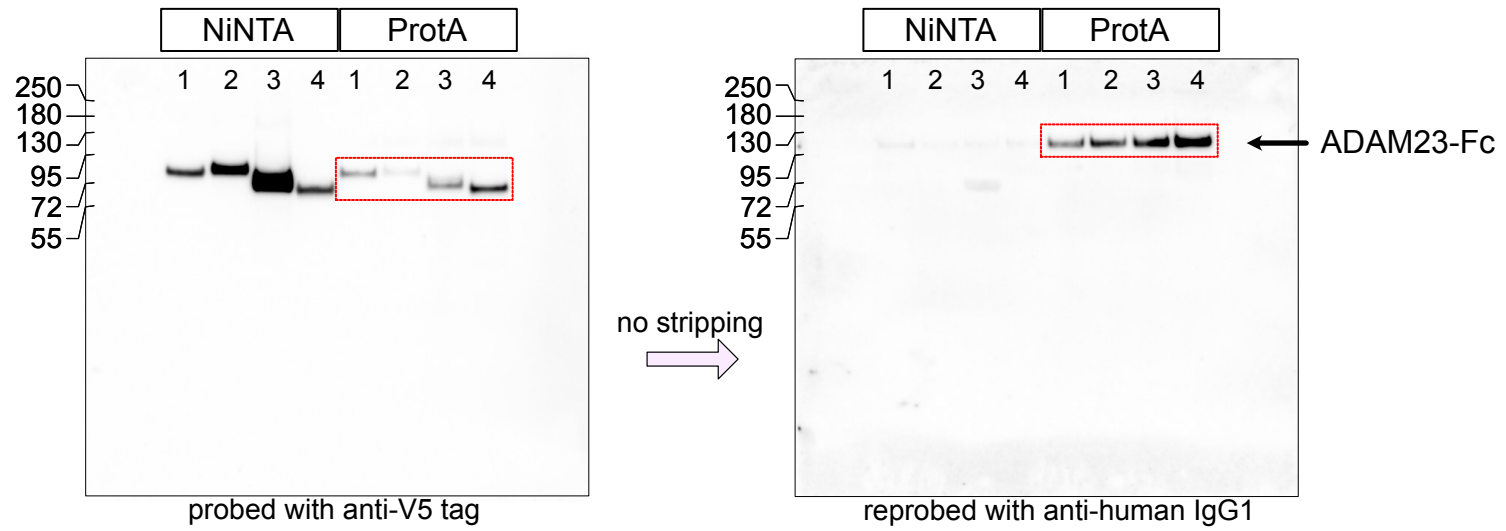

Supplement: SourceData F2 — is the source file for Fig. 2. [file JCB_202211031_SourceDataF2.pdf]

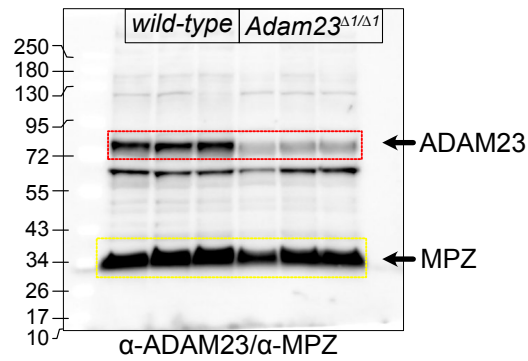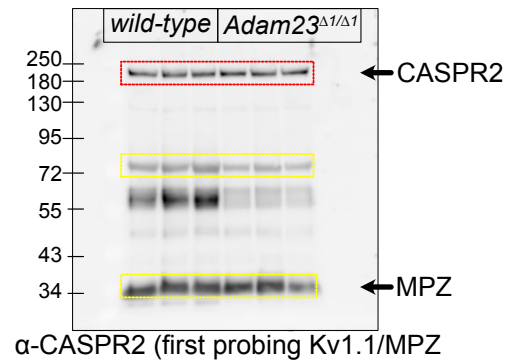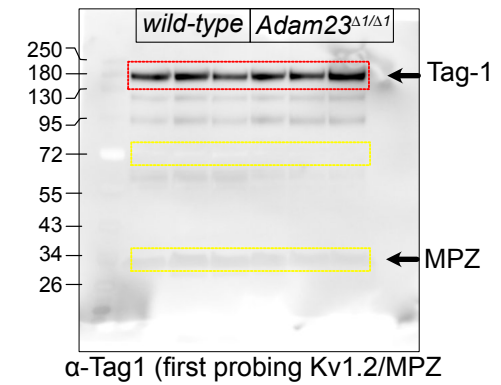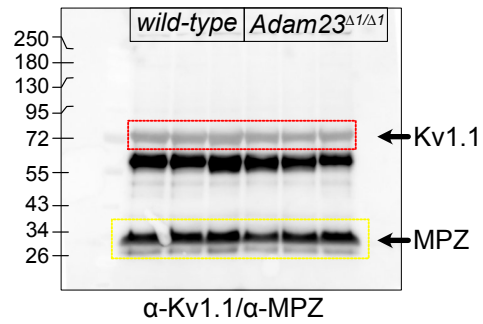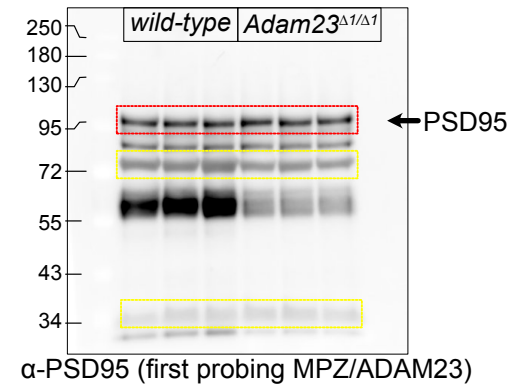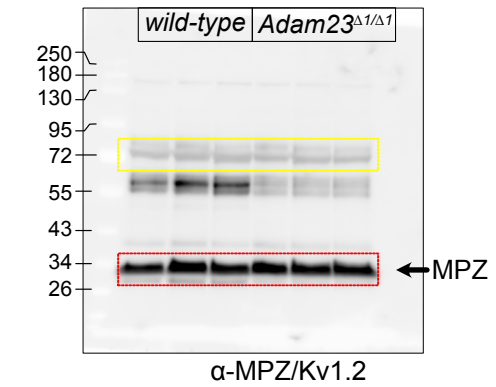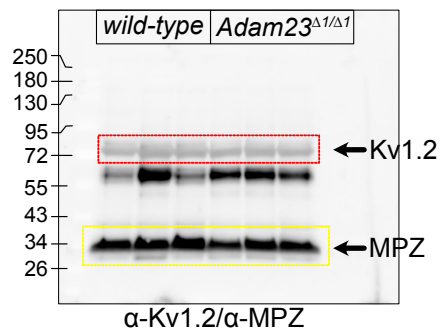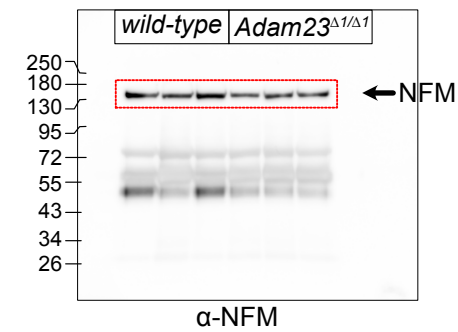

Source data Figure 5 Kozar-Gillen et al

Supplement: SourceData F5 — is the source file for Fig. 5. [file JCB_202211031_SourceDataF5.pdf]

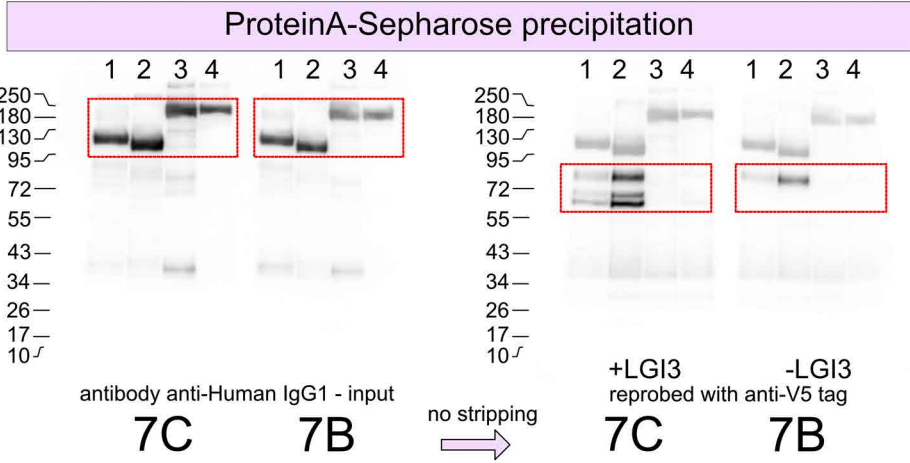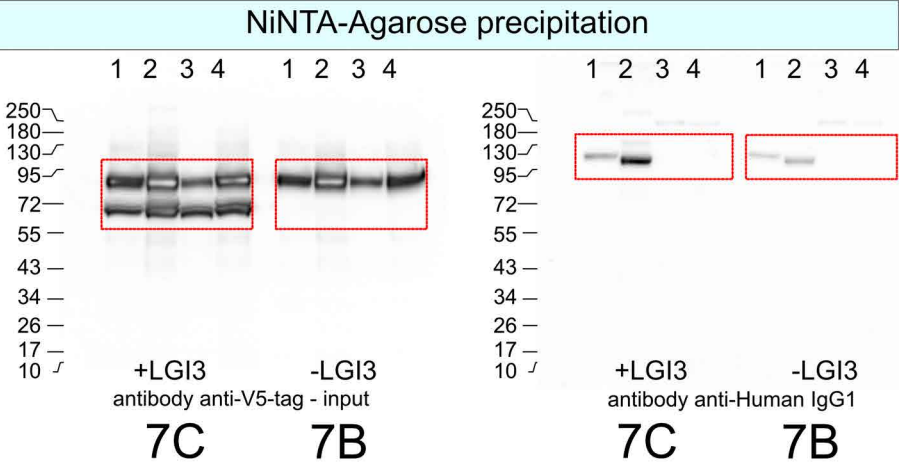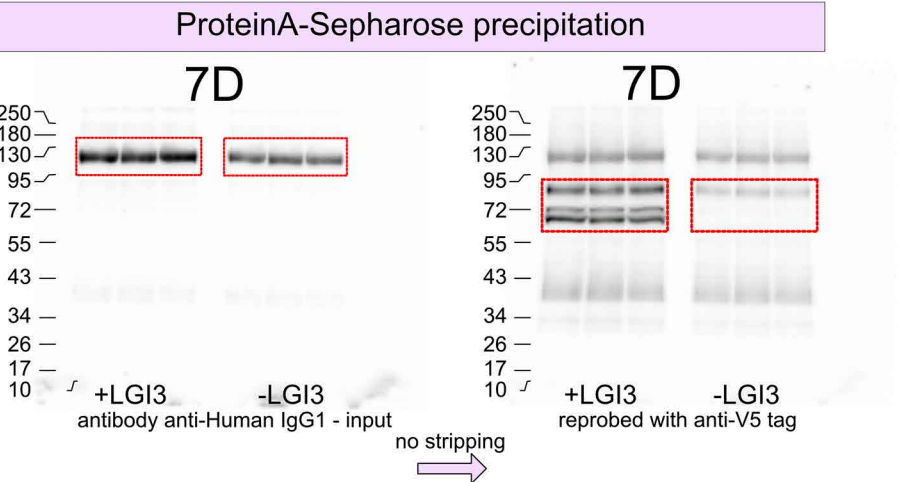

Source data Figure 7 Kozar-Gillen et al

Supplement: SourceData F7 — is the source file for Fig. 7. [file JCB_202211031_SourceDataF7.pdf]
